# Cytomegalovirus Retinitis in Newly Diagnosed Advanced HIV Infection: A Three-Case Series Emphasizing Multidisciplinary Infection Screening

**DOI:** 10.3390/idr18040066

**Published:** 2026-06-30

**Authors:** Shintaro Yataka, Kinya Tsubota, Kei Wakatsuki, Risa Sugawara, Masaki Asakage, Yoshihiko Usui

**Affiliations:** Department of Ophthalmology, Tokyo Medical University, 6-7-1 Nishi-shinjuku, Shinjuku-ku, Tokyo 160-0023, Japan

**Keywords:** cytomegalovirus retinitis, human immunodeficiency virus, coinfection, opportunistic infection, sexually transmitted infection, hepatitis B, syphilis, tuberculosis, retinal detachment

## Abstract

Background/Objectives: Cytomegalovirus (CMV) retinitis remains a significant opportunistic infection in patients with advanced human immunodeficiency virus (HIV) infection, particularly with late HIV diagnoses. This three-case series aimed to describe HIV-associated CMV retinitis in newly diagnosed advanced HIV infection with documented concurrent and/or prior infectious conditions, and to highlight the importance of bord systemic screening and multidisciplinary management. Methods: We retrospectively reviewed three male patients diagnosed with HIV-associated CMV retinitis at a tertiary ophthalmology referral center. Clinical findings, CD4-positive T-cell counts, HIV-RNA levels, aqueous humor CMV-DNA results, systemic infectious conditions, treatment and ocular outcomes were summarized. Results: All patients had marked cellular immunodeficiency, with CD4-positive T-cell counts ranging from 46 to 141 cells/µL, and CMV-DNA was detected in aqueous humor in all cases. The infectious burden was substantial: all three patients had syphilis and hepatitis B virus infection, two had oral candidiasis, and individual patients had chlamydia infection, tuberculosis, amebic colitis, or a history of herpes zoster. One patient was initially suspected of having syphilitic uveitis, which illustrates how coinfections may obscure the diagnosis of CMV retinitis. Retinal detachment occurred in two cases and was surgically repaired with anatomical recovery. Conclusions: These cases emphasize that CMV retinitis in newly diagnosed advanced HIV infection should prompt broad infection screening and multidisciplinary evaluation, particularly in the setting of delayed HIV diagnosis and severe immunosuppression. Comprehensive screening for opportunistic and sexually transmitted infections, prompt ocular virological confirmation, and multidisciplinary management are essential in patients with HIV-associated CMV retinitis.

## 1. Introduction

Cytomegalovirus (CMV) retinitis is a vision-threatening opportunistic infection that classically occurs in individuals with profound cellular immunodeficiency, particularly those with advanced human immunodeficiency virus (HIV) infection [1]. Despite a major decline in incidence in regions with broad access to antiretroviral therapy (ART), CMV retinitis continues to be diagnosed in patients with late HIV diagnosis, interrupted care, or delayed immune restoration [2,3]. Current guidelines for HIV-associated opportunistic infections emphasize that CMV retinitis remains the most common CMV end-organ disease in people with HIV [4].

The clinical management of CMV retinitis has become increasingly complex because it may coexist with other infectious diseases that share common epidemiologic routes, such as sexual transmission and blood exposure, or exploit common immunologic vulnerabilities, including tuberculosis, invasive fungal infections, and protozoal disease. In the Asia–Pacific region, a recent international consensus highlighted practical issues in CMV retinitis management, including first-line systemic antiviral therapy, discontinuation of maintenance therapy after immune reconstitution, and differentiation of immune recovery uveitis from CMV relapse [5]. However, reports specifically focusing on CMV retinitis as an ophthalmic presentation associated with multiple concurrent infections, and emphasizing the need for broad systemic screening at diagnosis, remain limited.

Herein, we describe three cases of HIV-associated CMV retinitis confirmed by aqueous humor CMV-DNA detection and complicated by documented concurrent or prior infectious conditions. These cases emphasize that CMV retinitis in newly diagnosed advanced HIV infection should prompt broad infection screening and multidisciplinary evaluation, rather than suggesting a CMV retinitis-specific infectious pattern.

## 2. Aqueous Humor PCR Analysis

The presumed causative virus was determined using PCR-based detection and quantification of herpesvirus DNA in aqueous humor samples. Aqueous humor samples of approximately 0.05–0.1 mL were collected using anterior chamber paracentesis under aseptic conditions. Qualitative and/or quantitative real-time PCR testing for CMV, HSV-1, HSV-2, and VZV was performed at an external reference laboratory (SRL, Japan) using the laboratory’s validated, quality-controlled protocol. When the aqueous humor volume was limited, the sample was diluted to a final volume of 2.0 mL before PCR analysis. In such cases, the viral copy number was corrected by multiplying the measured value by the dilution factor and was expressed as the estimated copy number in the original aqueous humor sample. PCR positivity was defined according to the laboratory’s detection threshold of ≥1.0 × 10^2^ copies/mL. The presumed causative virus was assigned based on PCR positivity, with priority given to the virus showing the dominant quantitative signal when quantitative data were available.

## 3. Case Presentation

This retrospective observational case series included three HIV-positive male patients diagnosed with CMV retinitis at an ophthalmology tertiary referral center. Clinical findings, laboratory results, aqueous humor CMV-DNA levels, systemic coinfections, treatments, and outcomes were reviewed. A summary of clinical and infectious characteristics is shown in Table 1.

### 3.1. Case 1

**Figure 1:**
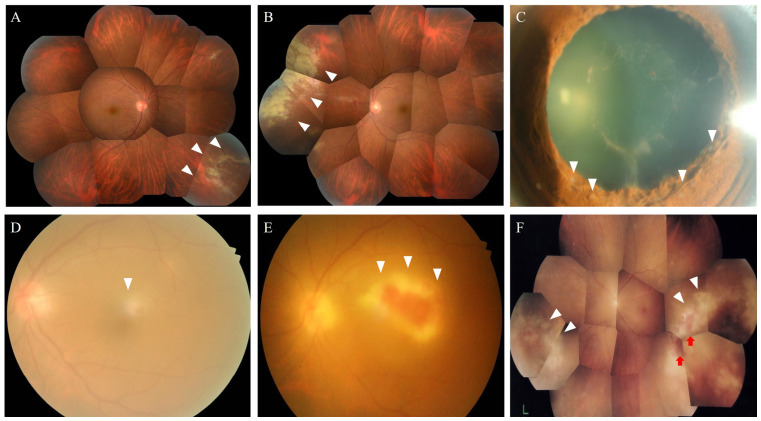
Clinical findings of three patients with HIV-associated CMV retinitis and concomitant infections. (**A**,**B**) Case 1: Fundus photographs of the right eye (**A**) and left eye (**B**) show peripheral yellow/white necrotizing retinitis lesions (arrowheads). In the right eye, a retinal tear with localized retinal detachment was also observed (arrowhead), indicating a structural complication of peripheral necrotizing retinitis. (**C**–**E**) Case 2: Slit lamp photograph of the left eye showing fibrin deposition and iris nodules ((**C**), arrowheads)—findings that initially suggested severe anterior uveitis. Fundus photograph at presentation showing a yellow/white exudative retinitis lesion involving the macula ((**D**), arrowheads), indicating a high risk of central visual impairment. One month later, enlargement of the macular lesion with increased retinal hemorrhage was observed ((**E**), arrowheads), prompting further evaluation for infectious retinitis and aqueous humor PCR testing. (**F**) Case 3: Fundus montage of the left eye shows peripheral yellow/white retinitis lesions (arrowheads), retinal hemorrhage (red arrows), and segmental retinal vasculitis, consistent with active CMV retinitis involving the peripheral retina.

A 42-year-old man presented with a 2-week history of bilateral floaters. Initial evaluation at another facility suggested bilateral uveitis and right retinal detachment, prompting referral to our center. Best-corrected visual acuity (BCVA) was 16/20 in the right eye and 18/20 in the left eye, and intraocular pressure was 11 mmHg OD and 12 mmHg OS. Slit lamp examination revealed 1+ anterior chamber cells bilaterally. Fundus examination showed white exudative lesions with a retinal break and localized retinal detachment in the right eye, with additional white lesions in the contralateral eye (Figure 1A,B). Systemic evaluation demonstrated advanced HIV infection, with a CD4-positive T-cell count of 63.9 cells/µL and HIV-1 RNA of 8.0 × 10^5^ copies/mL. The clinical record showed a history of hepatitis B virus (HBV) infection, syphilis, and chlamydia infection. Polymerase chain reaction (PCR) testing of aqueous humor detected CMV-DNA at 7.6 × 10^4^ copies/mL, supporting a diagnosis of CMV retinitis. Intravenous ganciclovir was initiated, and ART including agents with anti-HBV activity was started. Kaposi sarcoma developed during the follow-up in Case 1 and was recorded as an AIDS-defining malignancy. Three months after ART initiation, intraocular inflammation worsened, and aqueous humor PCR again detected CMV-DNA (5 × 10^4^ copies/mL). The clinical course was considered compatible with IRU/ocular IRIS with possible persistent or recurrent CMV activity. Intravenous foscarnet was administered, and a posterior sub-Tenon corticosteroid injection was subsequently performed for inflammatory control, resulting in improvement of intraocular inflammation. Surgical repair of progressive retinal detachment achieved retinal reattachment. At 1-year follow-up, no recurrent detachment was observed and BCVA was stable at 8/20 in both eyes.

### 3.2. Case 2

A 48-year-old man presented with impaired vision and haze in the left eye, was initially suspected of having syphilitic uveitis at another clinic, and was referred to our center for evaluation. BCVA was 20/20 in the right eye and 12/20 in the left eye. Fundus examination showed a yellow/white exudative lesion involving the macula, accompanied by fibrin formation and iris nodules in the left eye (Figure 1C,D). Laboratory tests revealed a CD4-positive T-cell count of 46 cells/µL and HIV-1 RNA of 1.4 × 10^5^ copies/mL, with positive test results for HBV and syphilis. Chest computed tomography demonstrated multiple pulmonary micronodules, and Mycobacterium tuberculosis was isolated from gastric fluid cultures. Oral candidiasis was also diagnosed. Syphilitic uveitis was initially suspected, and oral amoxicillin therapy was started. One month after the initial visit, enlargement of the macular exudates and an increase in retinal hemorrhage were observed (Figure 1E). Suspected CMV retinitis promoted the performance of aqueous humor PCR, showing CMV-DNA at 3.3 × 10^3^ copies/mL, and confirming CMV retinitis. Oral valganciclovir was initiated on day 36, followed by ART including agents with anti-HBV activity on day 104. Ocular inflammation improved with antiviral therapy. Three months after ART initiation, intraocular inflammation worsened, which was considered symptomatic of IRU/ocular IRIS. The patient was monitored closely with continued multidisciplinary management. Although syphilitic uveitis was initially suspected because of positive syphilis serology, the retinal lesion enlarged and retinal hemorrhage increased during initial oral amoxicillin therapy. After aqueous humor PCR detected CMV-DNA, amoxicillin was discontinued and anti-CMV therapy was initiated, resulting in improved ocular findings. Therefore, syphilis was regarded as a coexisting systemic infection, whereas CMV retinitis was considered the principal active ocular diagnosis. Nevertheless, because ocular syphilis has diverse clinical manifestations and lacks a single definitive ocular biomarker, a concomitant contribution of syphilitic ocular inflammation could not be completely excluded.

### 3.3. Case 3

A 43-year-old man presented with floaters in the left eye. BCVA was 2/20 in the right eye and 20/1000 in the left eye. Slit lamp examination revealed 1+ anterior chamber cells in the left eye. Fundus examination showed peripheral white exudative lesions with retinal hemorrhage and segmental vasculitis in the left eye (Figure 1F). Laboratory tests revealed a CD4-positive T-cell count of 141 cells/µL and HIV-1 RNA of 2.4 × 10^5^ copies/mL. A history of syphilis and herpes zoster was confirmed from the medical records, while HBV infection was newly identified upon laboratory testing. Aqueous humor PCR detected CMV-DNA at 2.9 × 10^3^ copies/mL. During systemic evaluation, oral candidiasis and amebic colitis were diagnosed. Intravenous ganciclovir was started on day 9, followed by ART on day 31. No clinical signs suggestive of IRIS or IRU, such as increased anterior chamber inflammation, vitritis, cystoid macular edema, or reactivation/worsening of retinitis after ART initiation, were observed during the follow-up. Retinal detachment developed but was surgically repaired with successful anatomical recovery.

Infection counts exclude CMV retinitis, which was present in all cases, and include HIV infection and documented systemic concurrent/active or prior infections.

## 4. Discussion

This case series illustrates that CMV retinitis may occur in patients with newly diagnosed advanced HIV infection who have documented concurrent or prior infectious conditions. All three patients exhibited profound cellular immunodeficiency, as indicated by markedly low CD4-positive T-cell counts. Because this report includes only three patients and has no control group, our findings should not be interpreted as demonstrating that CMV retinitis independently predicts multiple concurrent infections or represents a CMV retinitis-specific infectious pattern. Rather, these cases should be interpreted in the context of delayed recognition of advanced HIV infection, in which other opportunistic and sexually transmitted infections should be actively considered. This interpretation is supported by previous case–control evidence: Hodge et al. compared AIDS patients with and without CMV retinitis and reported that the number of previous opportunistic infections was independently associated with CMV retinitis, even after restricting the analysis to patients with CD4-positive T-cell counts below 50 cells/μL [6]. Previous nonocular CMV infection and previous Mycobacterium infection were also associated with CMV retinitis in that study [6]. Importantly, documented infections in the present retrospective case series included both concurrent/active infections and prior infection history; therefore, we separately summarized these categories in Table 1 and focused our interpretation primarily on concurrent/active infections. The median number of concurrent/active infectious conditions, including HIV infection, was three per patient (range, 1–4), whereas the median number of prior infections was two per patient (range, 1–3). Although not all patients had multiple concurrent active infections at presentation, all three had newly diagnosed advanced HIV infection and documented infectious conditions or prior infection history requiring systemic assessment. Therefore, the clinical message of this case series is not that CMV retinitis uniformly indicates multiple active infections, but that CMV retinitis in newly diagnosed advanced HIV infection should prompt broad infection screening and multidisciplinary evaluation.

Based on this clinical message, multidisciplinary collaboration in these cases involved several concrete clinical activities. Ophthalmologists established the ocular diagnosis, performed ocular fluid testing when necessary, evaluated the extent and activity of retinitis, and monitored ocular complications such as macular involvement and retinal detachment. When HIV infection was confirmed or strongly suspected, internists specializing in HIV care were consulted to evaluate systemic immune status and determine the timing and regimen of ART. Infectious disease specialists were also involved when concomitant infections, including syphilis and tuberculosis, were suspected during the diagnostic work-up. Treatment priorities were determined through discussion among these departments, particularly when multiple infections and multiple organ systems were involved. In addition, ophthalmic findings were used not only for diagnosis but also as indicators of treatment response and disease control during systemic management. These collaborative activities were particularly important because CMV retinitis in this series occurred in the setting of newly diagnosed advanced HIV infection, in which ocular disease activity needed to be integrated with systemic infectious evaluation and treatment prioritization.

CMV retinitis typically manifests as characteristic necrotizing retinitis with granular borders and hemorrhage [7]. However, in the context of multiple coinfections, clinical manifestations may be atypical or overlapping. Case 2 illustrates the diagnostic difficulty created by overlapping infections in advanced HIV infection. Ocular syphilis is known as a “great masquerader” because it can present as anterior, intermediate, posterior, or panuveitis, retinal vasculitis, optic nerve involvement, and other inflammatory phenotypes [8,9]. A diagnosis is generally based on compatible ocular findings together with reactive syphilis serology, and no single ocular biomarker definitively distinguishes syphilitic uveitis from other infectious retinitides. In our patient, positive syphilis serology initially led to suspicion of syphilitic uveitis. However, progression of the retinal lesion despite initial amoxicillin therapy, profound immunodeficiency due to newly diagnosed HIV infection, detection of CMV-DNA in aqueous humor, and subsequent improvement after anti-CMV therapy supported CMV retinitis as the principal active ocular diagnosis. This case does not indicate that syphilitic ocular involvement was definitively ruled out; rather, it emphasizes that CMV retinitis and syphilis may coexist in advanced HIV infection and that ocular fluid PCR can be crucial when the clinical course is atypical or progressive. Coinfections may alter inflammatory responses or obscure classic fundus findings, and microbiological confirmation by aqueous humor PCR becomes essential for accurate diagnosis [10,11].

Retinal detachment was observed in two of the three patients, and this high rate likely reflects advanced retinal necrosis and structural fragility associated with delayed presentation and severe immunosuppression. Retinal detachment remains one of the most serious complications of CMV retinitis, even in the ART era [12]. Prompt antiviral treatment combined with timely vitreoretinal surgical intervention is critical for anatomical preservation [13,14]. However, CMV-DNA load alone may not fully determine visual prognosis or retinal detachment risk [15]. In the present series, the highest aqueous CMV-DNA level was observed in Case 1, which had bilateral disease and retinal detachment, but Case 3 developed retinal detachment despite a lower CMV-DNA level. This discrepancy suggests that factors other than viral load, including lesion location, lesion size, activity of retinitis, and structural fragility of necrotic retina, are important. Previous clinical studies have reported that larger lesion size, bilateral disease, active retinitis, anterior lesion location, and retinal detachment are associated with poor ocular outcomes [16,17,18,19]. In addition, visual prognosis is strongly influenced by involvement of the central retina, particularly macular or Zone 1 lesions [15]. Therefore, aqueous CMV-DNA level should be interpreted as one component of severity assessment, together with anatomical lesion location, lesion extent, timing of diagnosis, and the presence or risk of retinal detachment. IRIS is another important consideration when ART is initiated in patients with advanced HIV infection and CMV retinitis [20,21,22]. In the eye, IRU represents an ocular form of IRIS and may manifest as anterior chamber inflammation, vitritis, cystoid macular edema, epiretinal membrane formation, or worsening ocular inflammation after immune recovery [20,21,22]. In the present series, Cases 1 and 2 developed worsening intraocular inflammation approximately 3 months after ART initiation, whereas Case 3 did not develop clinically diagnosed IRIS/IRU. Case 1 also had detectable aqueous CMV-DNA at the time of worsening inflammation, suggesting possible overlap between immune-mediated inflammation and persistent or recurrent CMV activity. These findings emphasize that worsening ocular inflammation after ART initiation should be evaluated for both IRU/ocular IRIS and active CMV retinitis, and that anti-CMV therapy and anti-inflammatory treatment should be individualized through close ophthalmic and systemic monitoring.

This study has several limitations. First, this was a small retrospective case series from a tertiary referral center. Such centers may disproportionately receive patients with severe ocular disease, atypical presentations of HIV/AIDS, and diagnostic dilemmas requiring specialized ophthalmic evaluation. Therefore, the infectious burden observed in this series may have been overestimated, and the frequency of severe ocular complications, including retinal detachment, may be higher than that expected in the broader population of patients with HIV-associated CMV retinitis. Second, because of the retrospective design, the exact timing of some prior infections may not be determined from the medical records, and prior infection history and concurrent/active infections may not be completely separated each time. These limitations preclude estimation of the true prevalence of concomitant infections or retinal detachment in newly diagnosed advanced HIV infection.

## 5. Conclusions

In patients with HIV-associated CMV retinitis, multiple concurrent or prior infections may complicate both diagnosis and management. Severe immunosuppression predisposes these individuals to overlap opportunistic infections, which may alter ocular presentation and delay appropriate therapy. Early virological confirmation, comprehensive systemic evaluation, and coordinated multidisciplinary care—including antiviral therapy, timely initiation of ART, and vitreoretinal surgery when required—are crucial for improving both ocular and systemic outcomes.

## Figures and Tables

**Table 1:** Summary of clinical characteristics, HIV-related outcomes, and infectious conditions in three patients with HIV-associated CMV retinitis.

Variable	Case 1	Case 2	Case 3	Summary
Age, years	42	48	43	Median 43 (range, 42–48)
Sex	Male	Male	Male	3 of 3
HIV stage classification	CDC stage 3 (AIDS)	CDC stage 3 (AIDS)	CDC stage 3 (AIDS)	3 of 3
CMV retinitis affected eye	Bilateral	Unilateral	Unilateral	1 bilateral; 2 unilateral
Aqueous humor CMV-DNA, copies/mL	7.6 × 10^4^	3.3 × 10^3^	2.9 × 10^3^	3 of 3
Anti-CMV treatment	IV ganciclovir 500 mg/day for 3 weeks, followed by oral valganciclovir 900 mg/day	Oral valganciclovir 1800 mg/day for 4 weeks, followed by 900 mg/day	IV ganciclovir 500 mg/day for 3 weeks, followed by oral valganciclovir 900 mg/day	Not applicable
Best corrected visual acuity at initiation of anti-CMV treatment (logMAR)	0.05	0.22	1.70	Median 0.22 (range, 0.05–1.7)
Best corrected visual acuity at final visit (logMAR)	0.40	0.70	2.00	Median 0.70 (range, 0.40–2.00)
Retinal detachment	Yes	No	Yes	2 of 3
Vitreoretinal surgery	Yes	No	Yes	2 of 3
CD4-positive T-cell count at HIV diagnosis, cells/μL	64	46	141	Median 64 (range, 46–141)
HIV-1 RNA at HIV diagnosis, copies/mL	8.0 × 10^5^	1.4 × 10^5^	2.4 × 10^5^	Median 2.4 × 10^5^ (range, 1.4–8.0 × 10^5^)
ART regimen	FTC/TAF + DTG	FTC/TDF + EFV	FTC/TDF + DTG	Not applicable
Time from HIV diagnosis to ART initiation (days)	32	104	31	Median 32 (range, 31–104)
CD4-positive T-cell count at 3 months after ART initiation, cells/μL	468	602	385	Median 468 (range, 385–602)
HIV-1 RNA at 6 months after ART initiation, copies/mL	<20	<20	<20	3 of 3 achieved viral suppression
IRIS/IRU during follow-up	Present; ocular inflammation worsened 3 months after ART initiation	Present; ocular inflammation worsened 3 months after ART initiation	Absent	2 of 3
Number of documented active infectious conditions, including HIV	1 (HIV)	4 (HIV, syphilis, oral candidiasis, tuberculosis)	3 (HIV, oral candidiasis, amebic colitis)	Median 3 (range, 1–4)
Number of prior infections	3 (HBV, syphilis, chlamydia infection)	1 (HBV)	2 (syphilis, HBV)	Median 2 (range, 1–3)
Total number of infections, including prior infections	4	5	5	Median 5 (range, 4–5)
Follow-up period, months	86	96	32	Median 86 (range, 32–96)

ART, antiretroviral therapy; CMV, cytomegalovirus; DTG, dolutegravir; EFV, efavirenz; FTC, emtricitabine; HBV, hepatitis B virus; IRIS, immune reconstitution inflammatory syndrome; IRU, immune recovery uveitis; IV, intravenous; TAF, tenofovir alafenamide; TDF, tenofovir disoproxil fumarate.

## Data Availability

The data are not publicly available because they contain potentially identifying clinical information. Deidentified data may be available from the corresponding author on reasonable request and with appropriate institutional approval.
